# An Electrophilic Uridine Building Block for Post‐Synthetic RNA Modification as Exemplified for Spin Labeling

**DOI:** 10.1002/chem.71319

**Published:** 2026-07-03

**Authors:** Lisa Ruetz, Sonja Leiner, Alessandro Marotto, Raphael Plangger, Christoph Kreutz

**Affiliations:** ^1^ Institute of Organic Chemistry and Center for Molecular Biosciences Innsbruck (CMBI) University of Innsbruck Innsbruck Austria

## Abstract

Inspired by the widely disseminated isothiocyanate (ITC) conjugation chemistry for proteins, we describe the synthesis of a 5‐isothiocyanato methyl‐modified uridine building block, which allows facile post‐synthetic modification of RNA with primary amines. The common concept in ITC labeling is based on an electrophilic labeling reagent, which is used to label reactive amino groups in the biomolecule. Here, we present the upside‐down approach—the RNA carries the electrophilic ITC functionality, which can, after solid‐phase synthesis, be modified with various labeling reagents equipped with a primary amino group. The 5‐pyrimidine labeling positions the unnatural tag in the major groove of the RNA and thus guarantees a minimal perturbation of the fold. The labeling procedure is exemplified for the 36 nucleotide (nt) stem loop C from the Moloney murine leukemia virus (MMLV) 5′‐Leader (SLC^A^ RNA) in a paramagnetic relaxation enhancement study using 4‐amino‐2,2,6,6‐tetramethyl‐1‐piperidine‐1‐oxyl (TEMPO‐NH_2_) as the labeling reagent.

## Graphical abstract

An electrophilic 5‐isothiocyanatomethyl uridine building block enables modular post‐synthetic RNA labeling with primary amine‐containing probes. Positioning the functionality in the major groove minimizes structural perturbation and allows facile conjugation of sensitive tags such as TEMPO spin labels for paramagnetic relaxation enhancement studies.

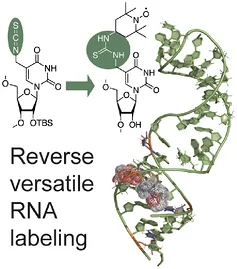

## 1 Introduction

Post‐synthetic modifications play an important role in expanding the structural and functional space of ribonucleic acid. Besides naturally occurring modifications, such as methylations and more complex modifiers (e.g., amino acids), oligonucleotide chemistry is a very well‐suited approach to allow the site‐specific introduction of a modification. A very recent example to post‐synthetically modify RNA is based on ribozymes, in which *in vitro* selection procedures yield catalytically active RNAs capable of modifying a target RNA with functionalities (e.g., alkyne groups) by using tailor‐made cofactors and to allow follow‐up chemistry [1, 2]. Another recent contribution is the use of aptamer domains to self‐modify the sequence with appropriate cofactors, for example, turn on fluorescence properties for *in vivo* RNA imaging [3]. These approaches rely on the synthesis of specialized cofactor analogues, such as *O* ^6^‐alkylguanine or synthetic, stabilized *S*‐adenosylmethionine (SAM) analogue cofactors.

Alternatively, if the modification is compatible with the reaction and deprotection conditions of RNA solid‐phase synthesis, a ribonucleotide phosphoramidite building block carrying the desired functionality can be synthesized and incorporated into the target RNA. This methodology is not feasible for sensitive modifiers, which are not stable during the acidic or oxidative conditions during RNA strand assembly or the strongly alkaline deprotection conditions in the RNA work‐up steps. Such instabilities were reported for certain fluorophores, such as cyanine, rhodamine, or BODIPY dyes. In these cases, the post‐synthetic approach is the method of choice. This approach is based on the synthesis of special phosphoramidites, which are then incorporated into a target RNA, and after deprotection and purification, the introduced functionalities can be used for post‐synthetic modification steps. Such well‐known modifiers are building blocks with nucleophilic primary amino groups, such as commercially available 5'‐ or 2'‐amino modifiers. Once the nucleophilic amino group is installed in the target RNA, a conjugation reaction using electrophilic *N*‐hydroxysuccinimide esters or cyanates/isothiocyanates (ITCs) can be carried out to tether the desired functionality. Another feasible strategy is based on the use of alkyne‐modified phosphoramidites, which allow the conjugation reaction by using click chemistry.

In nucleic acid NMR and EPR spectroscopy, a prominent modification is the introduction of radical spin labels to allow paramagnetic relaxation enhancement studies. Recent studies applied post‐synthetic attachment of radicals or used modified phosphoramidite precursors to site‐specifically introduce spin labels [4, 5, 6, 7, 8, 9, 10, 11, 12, 13, 14, 15, 16]. The advantage of the post‐synthetic conjugation of spin labels is the mild conditions during the labeling reaction, which leave the radical functionality intact. During RNA/DNA solid‐phase synthesis, the spin label is exposed to relatively harsh conditions, and especially the acidic conditions during the detritylation are reported to degrade the radical [17]. Facile spin labeling of RNA/DNA significantly expands the NMR toolbox for nucleic acids and can give unprecedented insight into biomolecular systems, such as DNA–protein complexes [18].

Here we report an inverted approach by using an electrophilic 5‐isothiocyanato methyl‐modified uridine building block, which can be post‐synthetically modified with primary amino group carrying reagents. The 5‐pyrimidine labeling proved to be minimally invasive as it positions the unnatural tag in the major groove of the RNA. The labeling procedure was exemplified for the 36 nucleotide (nt) stem loop C from the Moloney murine leukemia virus (MMLV) 5′‐Leader (SLC^A^ RNA) in a paramagnetic relaxation enhancement study (PRE) using 4‐amino‐2,2,6,6‐tetramethyl‐1‐piperidine‐1‐oxyl (TEMPO‐NH_2_) as the labeling reagent.

## 2 Results And Discussion

### 2.1 Synthetic Access to the 5‐ITC‐Methyl Uridine RNA Phosphoramidite **10** and Chemical Stability of the 5‐ITC‐Modified Uridine

The synthetic route to the ITC‐modified uridine building block **10** is shown below (Scheme 1). The route consists of nine steps and an overall yield of 9%, and in total 490 mg of the phosphoramidite **10** were obtained. In detail, starting from the sugar benzoylated 5‐hydroxymethyl uridine derivative **1** [19], the benzylic alcohol functionality was protected via a 4,4'‐dimethoxytrityl group (intermediate **2**), followed by the liberation of the sugar OHs, giving compound **3**. Then, the temporary 5',3'‐silylene and the 2'‐*O*‐*tert*.‐butyl‐dimethylsilyl (TBDMS) protecting groups were installed (compound **4**), setting the prerequisites for the transformation of the benzylic alcohol into an amino functionality. First, the OH group was set free (**5**), which was subsequently transformed into the pivaloyl ester **6**. The pivaloyloxy substituent was earlier used as a leaving group in post‐synthetic conversions of RNA to amino‐modified nucleic acids [20]. Here, we used the sterically demanding pivaloyloxy substituent to introduce the amino group, giving uridine derivative **7**. By using 7 M ammonia in a methanol solution and an elevated temperature (70°C), the nucleophilic substitution of the pivaloyl acid with the amino functionality could be achieved. Then, the classical amino to ITC conversion using thiophosgene was carried out with a very good yield (86%) to give compound **8** [21]. At this stage, the stability of the ITC‐modified uridine nucleoside **8** was tested. Small amounts (∼ 5 mg of **8** for each reagent solution) were incubated with the various reagent solutions used in the solid‐phase RNA synthesis procedure (detritylation solution: 1 mL 4% dichloroacetic acid in anhydrous toluene; activator solution: 1 mL 0.25 M *S*‐benzylthiotetrazole in anhydrous acetonitrile; capping A: 1 mL acetic anhydride/lutidine/anhydrous tetrahydrofuran 1/1/8; capping B: 1 mL anhydrous tetrahydrofuran/N‐methylimidazole 84/16; oxidation solution: 1 mL 0.2 M iodine in tetrahydrofuran/pyridine/water 7/2/1). Then, the stability of **8** was assessed using thin layer chromatography (TLC) after three time points (1, 15, and 30 min). A single TLC spot of **8** (R_f_ ca. 0.8, dichloromethane/methanol 95/5) was observed in all cases, indicating that **8** is stable under the reaction conditions of solid‐phase RNA strand assembly. In line with this observation, the ITC functionality also proved to be stable during the follow‐up reaction conditions. In a one‐pot procedure, first the 5',3'‐silylene protecting group was selectively removed at 0°C with a pyridine‐buffered hydrogen fluoride solution, and then the 5'‐(4,4'‐dimethoxytrityl) protecting group was attached to give compound **9** in 96% yield over two steps. Finally, the phosphoramidite functionality was introduced at the 3'‐hydroxyl in reasonable yield (51%) to give the desired RNA building block **10**.

**SCHEME 1:**
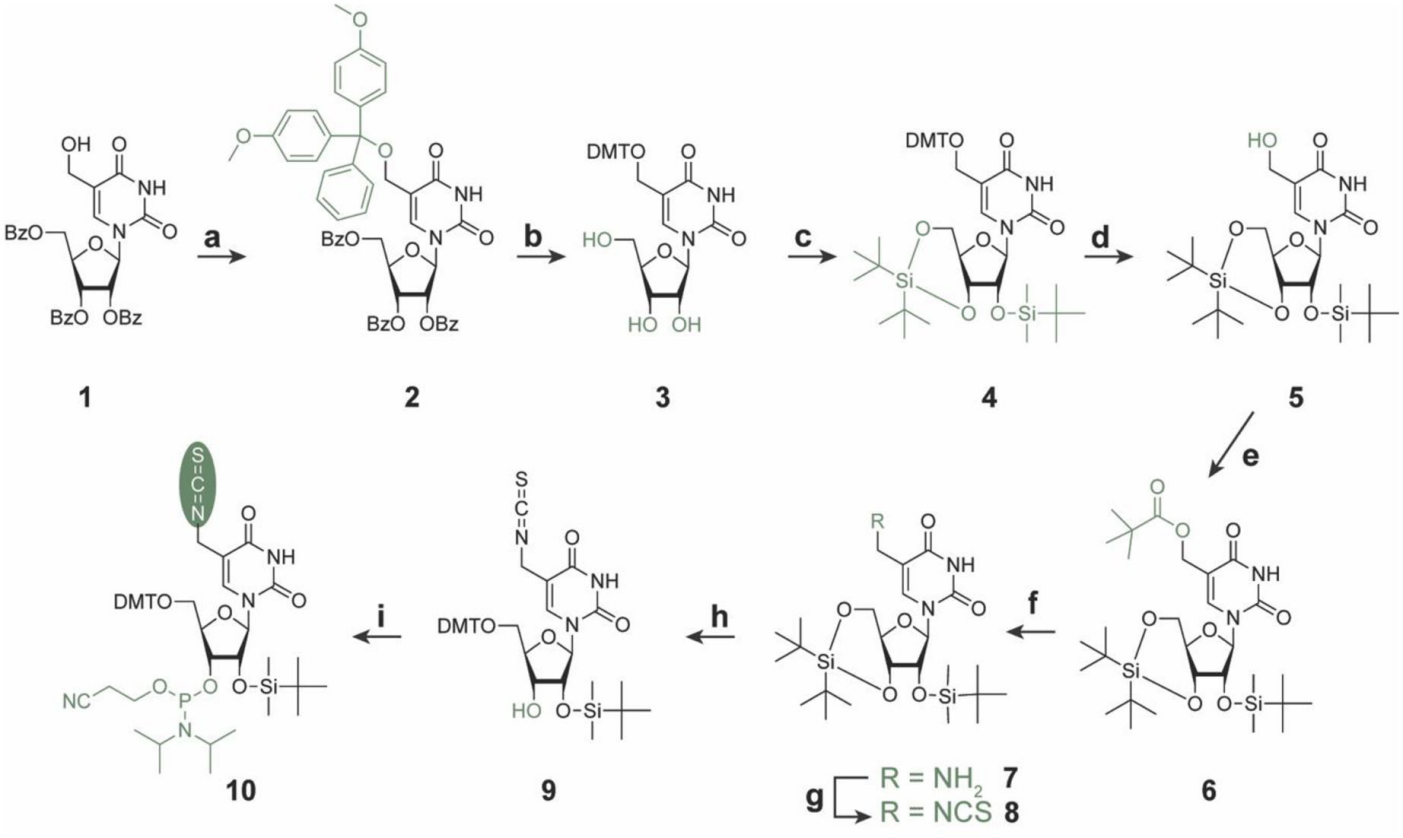
Synthetic route to 5‐isothiocyanatomethyl‐modified uridine phosphoramidite **10**. (a) DMT‐Cl in pyridine, DMAP_cat._, rt, 16 h, Ar, 95%. (b) 33% methylamine in ethanol, rt, 16 h, 95%. (c) (tBu)_2_Si(OTf)_2_, pyridine in DMF, 0°C, 4 h, Ar, then imidazole, TBDMS‐Cl, 60°C, 3 h, 95%. (d) dichloroacetic acid, pyridine in anhydrous dichloromethane, rt, Ar, 0.5 h, 56%. (e) pivaloyl chloride in pyridine, rt, Ar, 2.5 h, 94%. (f) 7 M ammonia in methanol, 70°C, 16 h, 51%. (g) thiophosgene in anhydrous chloroform, rt, 3 h, 86%. (h) HF·pyridine, pyridine in dichloromethane, 0°C, 4 h, Ar, then DMT‐Cl in pyridine, RT, 16 h, Ar, 96%. (i) CEP‐Cl, *N*,*N*‐diisopropylethylamine in anhydrous tetrahydrofuran, rt, 2 h, 51%.

### 2.2 Solid‐Phase RNA Synthesis of 5‐ITC‐U‐Modified RNA and Post‐Synthetic Labeling Procedure

With the encouraging chemical stability data of compound **8** in the presence of the solid‐phase reagent solutions, the 5‐ITC‐U building block **10** was used in the standard RNA synthesis setup, albeit with an increased coupling time (6 vs. 3 min, for standard building blocks). According to the trityl assay, the modified ITC uridine building block was incorporated with an efficiency comparable to the commercially available RNA phosphoramidites (98%–99%).

As the initial sequence, a 9 nt RNA (A_2_C_2_G_2_U_2_^ITC^U_1_) was chosen to optimize the modification and deprotection conditions. Initially, the standard procedure to remove the phosphate cyanoethyl groups—treatment of the solid support‐bound RNA with 20% diethylamine in acetonitrile for 10 min—was applied. This was followed by a treatment of the solid support‐bound RNA with 50 equivalents of TEMPO‐NH_2_ in ca. 1.5 mL anhydrous dimethylsulfoxide for 16 h at room temperature. The 50 equivalents are calculated with respect to the synthesis scale–that is, for a standard 1.3 µmol RNA synthesis, 65 µmol TEMPO‐NH_2_ are used. Finally, the standard alkaline deprotection (AMA: aqueous methylamine/aqueous ammonia, 37°C for 5 h) and the 2'‐protecting group removal (triethylamine trihydrofluoride in dimethylsulfoxide, 37°C, 16 h) were applied, and the crude RNA was analyzed by anion‐exchange high‐performance liquid chromatography (AIEX‐HPLC). A single major peak was observed, which was subsequently analyzed by liquid chromatography electron spray ionization mass spectrometry (LC‐ESI‐MS). The correct mass was not found; instead, the major component of the crude RNA solution was the diethylthiourea uridine derivative (Figure S3A).

Although considered a sterically hindered amino base, the diethylamine treatment leads to the undesired formation of the respective diethylthiourea derivative. Thus, in the next step, the diethylamine step was omitted, and the solid support‐bound fully protected RNA was directly subjected to the modifying reagent (TEMPO‐NH_2_) under the same conditions as before, and then the alkaline and fluoride deprotection conditions were applied. With this protocol, the major species in the AIEX chromatogram was the desired modified 9 nt RNA (Figure S3B). The two work‐up protocols are summarized (Scheme 2). With the optimized modification/deprotection protocol, variants of the larger 36 nt stem loop C from the Moloney Murine Leukemia Virus (MMLV) 5′‐Leader (SLC^A^ RNA) with stable isotope labeled residues and the 5‐ITC‐U modification at two positions (U16 and U31) were produced. These RNAs were again modified with the TEMPO‐NH_2_ reagent to allow paramagnetic relaxation enhancement (PRE) studies by NMR. The sequence, stable isotope, and 5‐ITC‐U labeling pattern, and the analytical data for the two 36 nt RNAs are shown (Figure 1A–C). Both RNAs were prepared according to the protocol developed and established for the 9 nt RNA.

**SCHEME 2:**
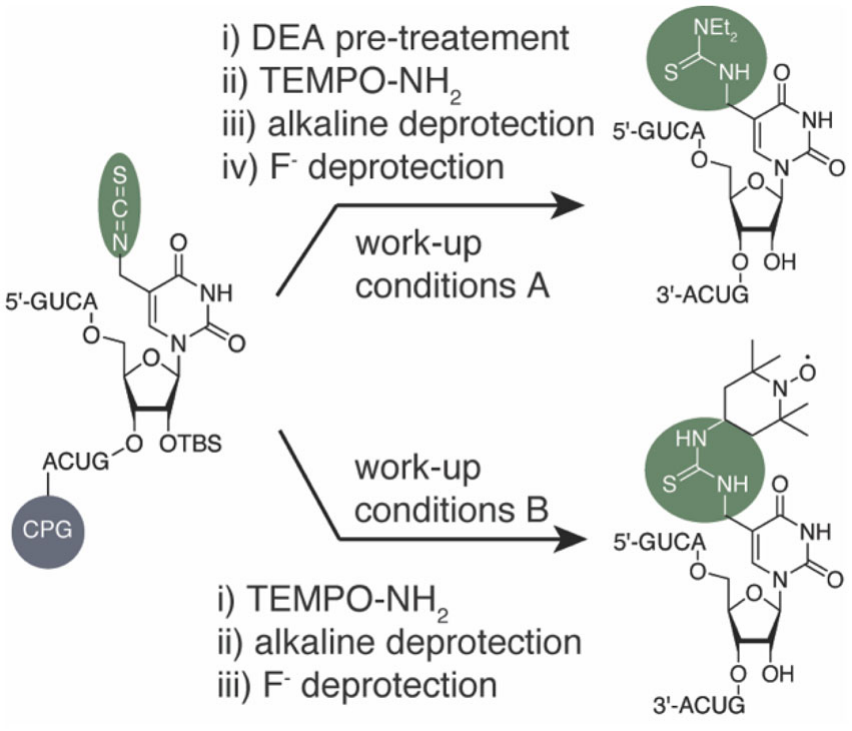
Optimization of work‐up and modifications procedures for 5‐ITC‐U modified RNA. *Work‐up conditions A*: (i) Treatment of solid support‐bound RNA with 50 mL 20% diethylamine (DEA) in acetonitrile for 10 min at rt, then wash with 3 × 20 mL CH_3_CN; (ii) 50 eq. TEMPO‐NH_2_ dissolved in DMSO, rt, 16 h. (iii) 1 mL aqueous ammonia (28%) and 1 mL aqueous methylamine (40%), 37°C, 3 h. (iv) Triethylamine trihydrofluoride in DMSO, 37°C, 16 h. *Work‐up conditions B*: same as conditions in A, but step (i) is omitted.

**FIGURE 1:**
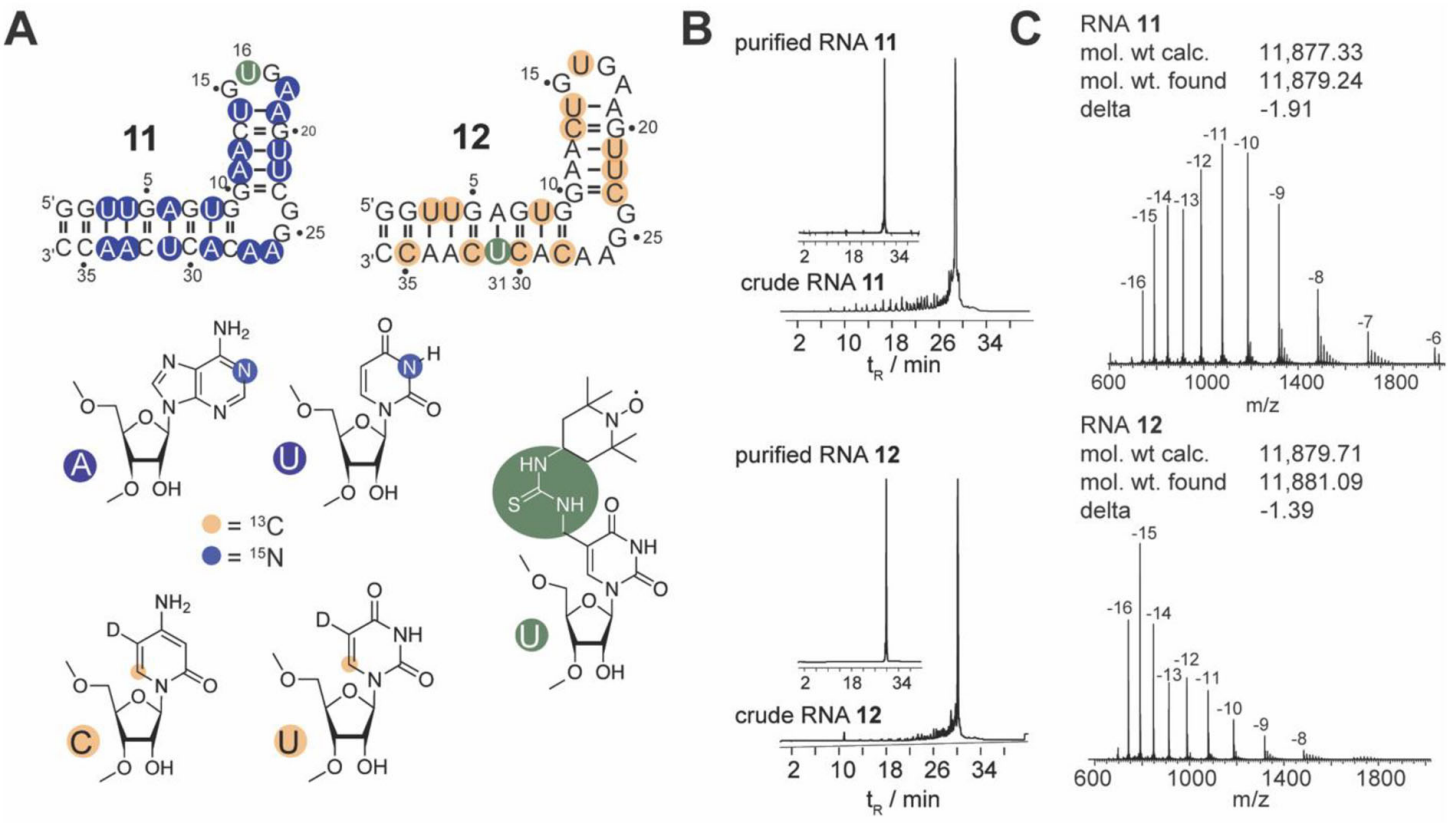
Analytical data of TEMPO radical tagged 36 nt SLC^A^ RNA **11** and **12** by using the 5‐ITC‐U labeling approach. (A) Stable isotope and TEMPO labeling pattern of the two SLC^A^ constructs **11** and **12** used in the PRE NMR study. (B) AIEX HPLC chromatograms of crude and purified 36 nt RNAs **11** and **12**. (C) ESI‐MS data and calculated masses of the 36 nt RNAs **11** and **12**.

The spin labeling positions and stable isotope labeling patterns were chosen to illustrate the versatility of the novel post‐synthetic conjugation approach. In RNA **11,** the 5‐ITC‐modified uridine was placed in the GUGA tetraloop at position 2 together with 1,3‐^15^N_2_‐uridines and 1–^15^N‐adenosines. In contrast, in RNA **12**, the TEMPO tag was placed in the major groove of the lower helix by replacing U31 with ^ITC^U, and the spin labeling was combined with 5‐D‐6‐^13^C‐pyrimidine labeling.

### 2.3 PRE NMR of TEMPO Tagged SLC^A^ RNAs

After the successful production of the spin and stable isotope‐labeled RNA **11** and **12,** the oligonucleotides were used in a paramagnetic relaxation enhancement (PRE) NMR study. First, the ^15^N‐labeled A and U residues in RNA **11** were used to observe the PRE effect exerted by the NO radical in position 16 (Figure 2A). The imino proton spectrum of construct **11** shows only minor differences between the active and quenched TEMPO state (Figure 2B), with only one significant PRE effect for G20. The imino proton resonance of U14 was not observed at room temperature, very likely due to the slightly destabilizing effect of the tag at position 16. No significant PRE effect was observed in the ^1^J_NH_ SOFAST correlation spectra of **11**. The potential effect on U21 is masked by an overlap with the U3 resonance (Figure 2C).

**FIGURE 2:**
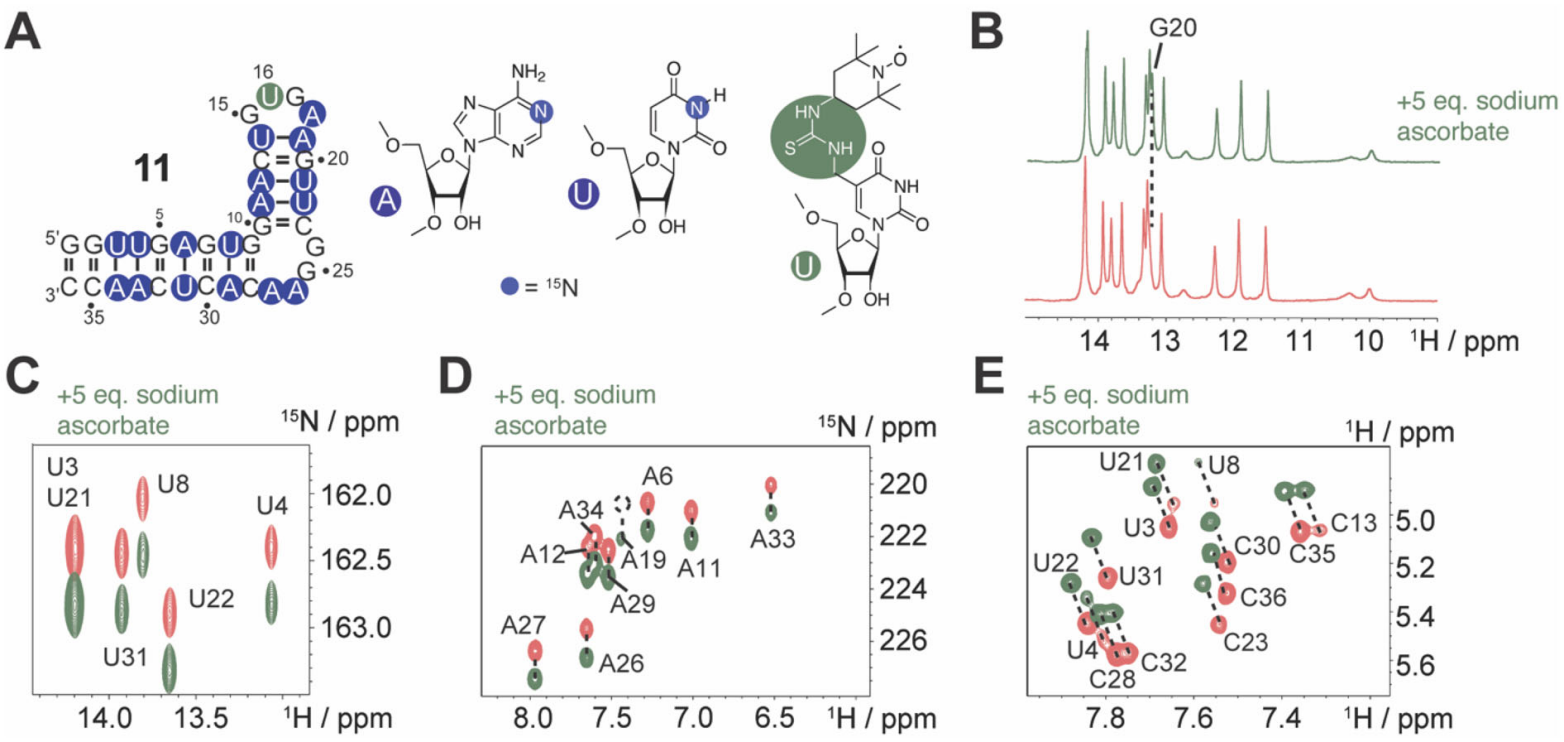
PRE NMR data of TEMPO radical tagged 36 nt SLC^A^ RNA **11**. (A) Stable isotope and TEMPO labeling pattern of the SLC^A^ construct **11**. (B) ^1^H NMR spectra with ^15^N decoupling and focus on the imino proton region before (red trace) and after (green trace) radical quench with sodium ascorbate. A significant PRE effect was observed for the G20 imino resonance. (C) ^1^H‐^15^N‐SOFAST NMR spectra with the same color coding as in B. (D) Long‐range ^1^H‐^15^N‐SOFAST NMR spectra with H2‐^15^N1 adenine correlations (same color coding as in B). (E) ^1^H‐^1^H‐TOCSY spectra with the same color coding as in B. *Note*: The para/diamagnetic spectra are shifted in the direct and/or indirect dimension to easily observe the PRE effect.

Then, the long‐range correlation between H2 and ^15^N1 in adenosines was used for the PRE readout (Figure 2D). Here, significant PRE effects were found for residues A11, A12, A26, and A27. Notably, the long‐range resonance for A18 was not observed in the diamagnetic state. This resonance is very likely exchange broadened beyond detection, as a similar behavior is found for A19. In the paramagnetic state, the A19 H2‐^15^N1‐ correlation peak was not observed (Figure 2D, dashed circle) as the bleaching PRE effect is very pronounced during the long INEPT delay (30 ms) of the long‐range correlation experiment. Upon the addition of 5 equivalents of ascorbic acid, the A19 long‐range resonance is rescued and found in the spectrum, but still shows significant line broadening, which could originate from the TEMPO modification at position 16. The TEMPO tag could introduce intermediate exchange between alternative loop conformations, leading to substantial line broadening. Finally, the PRE effect was probed by running a ^1^H‐^1^H‐TOCSY experiment and focusing on the H5‐H6‐pyrimidine region (Figure 2E). Again, significant PRE effects were observed, this time for C13 and U21. The TOCSY data further support an exchange process on the intermediate chemical shift time scale in the loop region, as the U14 H5‐H6 correlation peak could not be observed. A semi‐quantitative analysis of the PRE effect in RNA **11** for the long‐range H2‐^15^N1‐A and H5‐H6 TOCSY resonances by plotting their intensity ratios of the paramagnetic and diamagnetic state is shown in the supporting information (Figure S4A,B).

In RNA **12**, the TEMPO tag resides in the lower helix at U31 and was combined with 5‐D‐6‐^13^C‐pyrimidine labeling of all U and C residues in the SLC^A^ RNA (Figure 3A). By running ^1^H‐^13^C‐HSQC spectra in the para‐ and diamagnetic state, a qualitative PRE effect could be obtained (Figure 3B). To obtain a quantitative description of the PRE effect, the labeling scheme was used to determine H6 proton transverse relaxation rates to give the PRE rate ^1^H‐Γ_2_. Reliable ^1^H‐Γ_2_ rates could be determined for residues U8, C35, and U4 as described earlier [22]. For U4, the strongest PRE effect was found with a ^1^H‐Γ_2_ rate of ca. 32 s^−1^, which was translated into a distance of 15.5 Å [23]. The correlation time was calculated with the aid of HYDRONMR [24] and the reported NMR structure of SLC^A^ [25]. The distance is in very good agreement with a modeled TEMPO‐tagged SLC^A^ structure (15.5 Å calculated vs. 15 Å modeled). For U8 (calc. 21 Å vs. modeled 20 Å) and C35 (calc. 18 Å versus modeled 17 Å), similar results were obtained (Figure S5).

**FIGURE 3:**
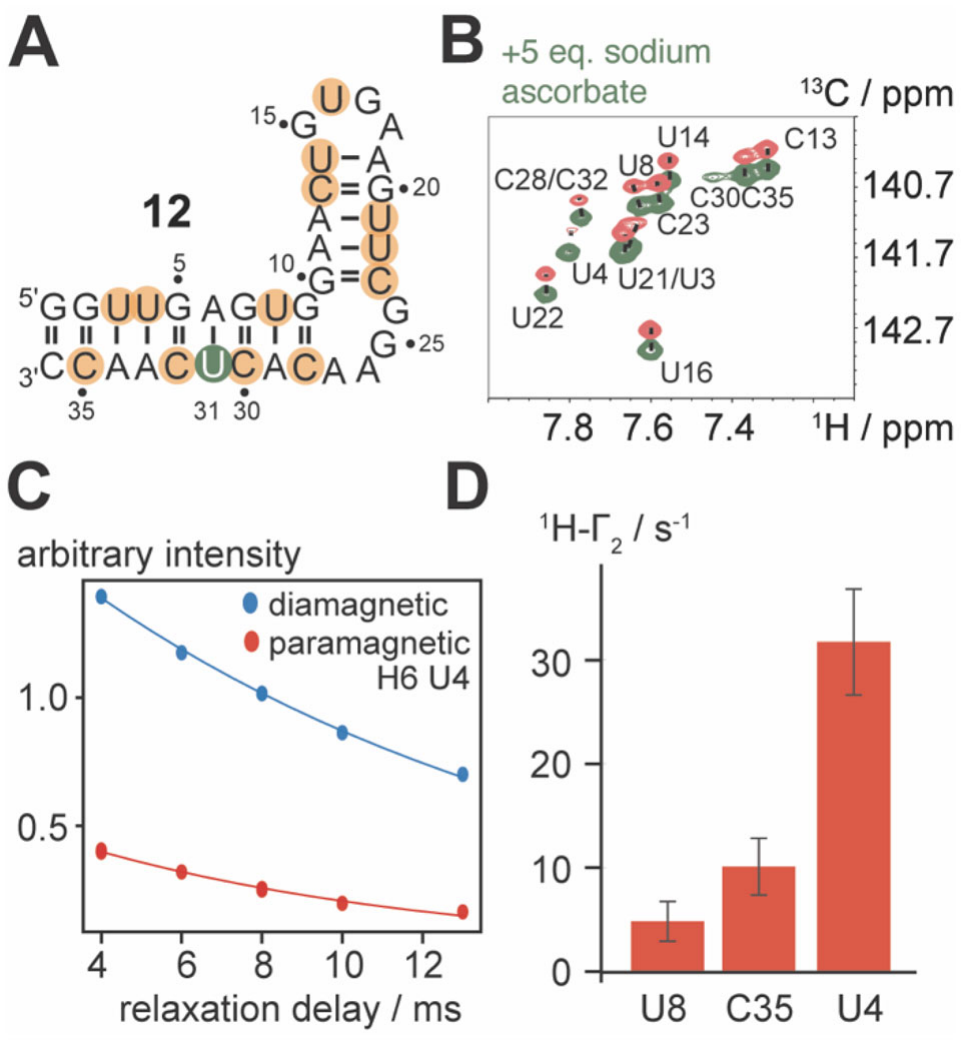
Quantitative determination of PRE effect in the 36 nt SLC^A^ RNA **12**. (A) Stable isotope and TEMPO labeling pattern of the SLC^A^ construct **12**. (B) ^1^H‐^13^C HSQC spectrum of **12** before (red) and after (green) radical quench with sodium ascorbate. *Note*: The paramagnetic spectrum is shifted in the indirect dimension to easily observe the PRE effect. (C) Exponential fit of ^1^H transverse relaxation rate experiment for residue U4 in para‐ and diamagnetic state. (D) H6 PRE rates (^1^H‐Γ_2_) of U8, C35 and U4 of RNA **12**.

These two examples nicely illustrate the effective spin‐labeling procedure relying on the novel 5‐ITC‐modified uridine building block **10**. The PRE effect can be determined in unlabeled samples by running homonuclear TOCSY‐type experiments, but also by relying on ^13^C/^15^N stable isotope labeling, which will be especially beneficial for larger RNA (> 50 nt) or if quantitative PRE rates are needed for long‐distance restraints, as shown for RNA **12**.

## 3 Conclusions

The work establishes an upside‐down approach by introducing an electrophilic 5‐ITC‐U RNA building block, which can be used for post‐synthetic modifications after solid‐phase synthesis of the target nucleic acid. The oligonucleotide can be site‐specifically modified with labels equipped with a primary amino functionality, which react with the solid support‐bound RNA to give a stable thiourea linkage. The applicability of the novel approach was exemplified in a paramagnetic relaxation enhancement NMR study of the SLC^A^ RNA by using TEMPO‐NH_2_ as the labeling reagent. A simple conjugation protocol starting with a treatment of the 5‐ITC‐U‐modified RNA on the solid support with an excess of labeling reagent (e.g., 50 eq. of TEMPO‐NH_2_ dissolved in DMSO, 16 h, rt), followed by the standard alkaline and fluoride deprotection steps, gave quantitative spin labeling as confirmed by mass spectrometry. Two variants of the SLC^A^ RNA with ^15^N and ^13^C labeling and two spin label positions were produced, and high‐quality PRE data could be obtained, which allowed a quantitative description of the paramagnetic relaxation enhancement effect to give long‐distance restraints. The facile and rather noninvasive spin labeling procedure places the radical tag into the major groove of the oligonucleotide if placed in a double helix. Thus, the fold of the RNA is only slightly perturbed, as exemplified for RNA **12**, and the labeling procedure can be regarded as a viable alternative to the widely disseminated approach based on the conjugation reaction of MTSL and 4‐thiouridine [26].

We foresee potential application of the RNA PRE labeling for the analysis of protein sliding mechanisms on RNA [27] by NMR, as earlier reported for DNA [18]. Further, the approach can also be used to introduce two TEMPO labels at the same time (Figure S6). Thereby, distance measurements via EPR spectroscopy can be obtained, which can be used in integrative structural biology approaches [28, 29, 30].

The novel 5‐ITC U building block can be further used in a tagging reaction for other applications if the labeling reagent offers a primary amino group. Such applications could include novel fluorescence labels but also the attachment of purification tags. The ITC functionality can also be used for the conjugation of synthetic peptides with a primary amino group to enhance cellular delivery or improve stability and pharmacokinetic properties.

## Funding

This research was funded in whole or in part by the Austrian Science Fund (FWF) [10.55776/F80 and 10.55776/PAT4733025 to CK] and the Austrian Research Promotion Agency FFG [West Austrian Bio‐NMR 858017 to CK].

## Conflicts of Interest

CK is an advisor and holds an ownership interest in Innotope GmbH, a company providing RNA stable isotope labeling products.

## Supporting information




**Supporting File**: Details on the synthetic procedures, RNA synthesis, NMR spectroscopy, and mass spectrometry; MS data of 9 nt RNA; Intensity ratios paramagnetic versus diamagnetic of RNA **11** for qualitative PRE analysis; Structure model of TEMPO tagged RNA **12** with distances to U4, U8, and C35; Analytical, NMR, and EPR data of double TEMPO tagged RNA. The authors have cited additional references within the Supporting Information [19, 22, 31, 32].

## Data Availability

The NMR data and Python fitting scripts are available (UIBK data repository, DOI: https://doi.org/10.48323/fmkhq‐xf509).

## References

[1] T. Okuda , A.‐K. Lenz , F. Seitz , J. Vogel , and C. Höbartner , “A SAM Analogue‐Utilizing Ribozyme for Site‐Specific RNA Alkylation in Living Cells,” Nature Chemistry 15 (2023): 1523–1531, 10.1038/s41557-023-01320-z. PMC10624628 37667013

[2] H.‐A. Chen , T. Okuda , A.‐K. Lenz , C. P. M. Scheitl , H. Schindelin , and C. Höbartner , “Structure and Catalytic Activity of the SAM‐Utilizing Ribozyme SAMURI,” Nature Chemical Biology 22 (2026): 938–947, 10.1038/s41589-024-01808-w. PMC13226088 39779902

[3] R. Bereiter , L. Flemmich , K. Nykiel , et al., “Engineering Covalent Small Molecule–RNA Complexes in Living Cells,” Nature Chemical Biology 21 (2025): 843–854, 10.1038/s41589-024-01801-3. PMC12122380 39762536

[4] E. C. Cetiner , H. R. A. Jonker , C. Helmling , et al., “Paramagnetic‐Iterative Relaxation Matrix Approach: Extracting PRE‐Restraints From NOESY Spectra for 3D Structure Elucidation of Biomolecules,” Journal of Biomolecular NMR 73 (2019): 699–712, 10.1007/s10858-019-00282-0. 31606877

[5] K. A. Schnorr , D. B. Gophane , C. Helmling , et al., “Impact of Spin Label Rigidity on Extent and Accuracy of Distance Information From PRE Data,” Journal of Biomolecular NMR 68 (2017): 53–63, 10.1007/s10858-017-0114-9. 28500543

[6] C. Helmling , I. Bessi , A. Wacker , et al., “Noncovalent Spin Labeling of Riboswitch RNAs To Obtain Long‐Range Structural NMR Restraints,” ACS Chemical Biology 9 (2014): 1330–1339, 10.1021/cb500050t. 24673892

[7] C. H. Wunderlich , R. G. Huber , R. Spitzer , K. R. Liedl , K. Kloiber , and C. Kreutz , “A Novel Paramagnetic Relaxation Enhancement Tag for Nucleic Acids: A Tool to Study Structure and Dynamics of RNA,” ACS Chemical Biology 8 (2013): 2697–2706, 10.1021/cb400589q. PMC3870906 24053726

[8] C. Höbartner , G. Sicoli , F. Wachowius , D. B. Gophane , and S. T. Sigurdsson , “Synthesis and Characterization of RNA Containing a Rigid and Nonperturbing Cytidine‐Derived Spin Label,” Journal of Organic Chemistry 77 (2012): 7749–7754, 10.1021/jo301227w. 22881715

[9] S. A. Shelke and S. T. Sigurdsson , “Noncovalent and Site‐Directed Spin Labeling of Nucleic Acids,” Angewandte Chemie International Edition 49 (2010): 7984–7986, 10.1002/anie.201002637. 20845340

[10] T. E. Edwards and S. T. Sigurdsson , “Site‐Specific Incorporation of Nitroxide Spin‐Labels Into 2′‐Positions of Nucleic Acids,” Nature Protocols 2 (2007): 1954–1962, 10.1038/nprot.2007.273. 17703207

[11] I. Krstić , R. Hänsel , O. Romainczyk , J. W. Engels , V. Dötsch , and T. F. Prisner , “Long‐Range Distance Measurements on Nucleic Acids in Cells by Pulsed EPR Spectroscopy,” Angewandte Chemie International Edition 50 (2011): 5070–5074. 10.1002/anie.201100886 21506223

[12] L. Büttner , J. Seikowski , K. Wawrzyniak , A. Ochmann , and C. Höbartner , “Synthesis of Spin‐Labeled Riboswitch RNAs Using Convertible Nucleosides and DNA‐Catalyzed RNA Ligation,” Bioorganic & Medicinal Chemistry 21 (2013): 6171–6180. 10.1016/j.bmc.2013.04.007 23664496

[13] A. Ramos and G. Varani , “A New Method To Detect Long‐Range Protein−RNA Contacts: NMR Detection of Electron−Proton Relaxation Induced by Nitroxide Spin‐Labeled RNA,” Journal of the American Chemical Society 120 (1998): 10992–10993, 10.1021/ja982496e.

[14] F. Kaiser , B. Endeward , A. Collauto , U. Scheffer , T. F. Prisner , and M. W. Göbel , “Spin‐Labeled Riboswitch Synthesized From a Protected TPA Phosphoramidite Building Block,” Chemistry – A European Journal 28 (2022): e202201822. 10.1002/chem.202201822 PMC9804336 35903916

[15] T. Weinrich , E. A. Jaumann , U. M. Scheffer , T. F. Prisner , and M. W. Göbel , “Phosphoramidite Building Blocks With Protected Nitroxides for the Synthesis of Spin‐Labeled DNA and RNA,” Beilstein Journal of Organic Chemistry 14 (2018): 1563–1569, 10.3762/bjoc.14.133. PMC6036967 30013683

[16] T. Weinrich , E. A. Jaumann , U. Scheffer , T. F. Prisner , and M. W. Göbel , “A Cytidine Phosphoramidite With Protected Nitroxide Spin Label: Synthesis of a Full‐Length TAR RNA and Investigation by In‐Line Probing and EPR Spectroscopy,” Chemistry—A European Journal 24 (2018): 6202–6207, 10.1002/chem.201800167. 29485736

[17] E. G. Rozantsev and V. D. Sholle , “Synthesis and Reactions of Stable Nitroxyl Radicals II. Reactions 1,” Synthesis 1971 (1971): 401–414, 10.1055/s-1971-21749.

[18] J. Iwahara and G. M. Clore , “Detecting Transient Intermediates in Macromolecular Binding by Paramagnetic NMR,” Nature 440 (2006): 1227–1230, 10.1038/nature04673. 16642002

[19] X. Yan , H. Sun , C. Mo , et al., “Design and Evaluation of Novel Pyrimidine‐Based Nucleoside Analogs as Antivirals: Identification of MCH‐1623 as a Potent Broad‐Spectrum Antiviral Candidate,” Bioorganic Chemistry 170 (2026): 109472, 10.1016/j.bioorg.2026.109472. 41512356

[20] K. Bartosik , E. Sochacka , and G. Leszczynska , “Post‐Synthetic Conversion of 5‐Pivaloyloxymethyluridine Present in a Support‐Bound RNA Oligomer Into Biologically Relevant Derivatives of 5‐Methyluridine,” Organic & Biomolecular Chemistry 15 (2017): 2097–2103, 10.1039/C6OB02674E. 28217770

[21] S. Saha , A. P. Jagtap , and S. T. Sigurdsson , Methods in Enzymology, ed. P. Z. Qin and K. Warncke (Academic Press, 2015), 397–414. 10.1016/bs.mie.2015.07.017 26478493

[22] J. Iwahara , C. D. Schwieters , and G. M. Clore , “Ensemble Approach for NMR Structure Refinement Against 1 H Paramagnetic Relaxation Enhancement Data Arising From a Flexible Paramagnetic Group Attached to a Macromolecule,” Journal of the American Chemical Society 126 (2004): 5879–5896, 10.1021/ja031580d. 15125681

[23] J. L. Battiste and G. Wagner , “Utilization of Site‐Directed Spin Labeling and High‐Resolution Heteronuclear Nuclear Magnetic Resonance for Global Fold Determination of Large Proteins With Limited Nuclear Overhauser Effect Data,” Biochemistry 39 (2000): 5355–5365, 10.1021/bi000060h. 10820006

[24] J. G. de la Torre , M. L. Huertas , and B. Carrasco , “HYDRONMR: Prediction of NMR Relaxation of Globular Proteins From Atomic‐Level Structures and Hydrodynamic Calculations,” Journal of Magnetic Resonance 147 (2000): 138–146. 10.1006/jmre.2000.2170 11042057

[25] J. Marchant , A. Bax , and M. F. Summers , “Accurate Measurement of Residual Dipolar Couplings in Large RNAs by Variable Flip Angle NMR,” Journal of the American Chemical Society 140 (2018): 6978–6983, 10.1021/jacs.8b03298. PMC6021016 29757635

[26] P. Z. Qin , K. Hideg , J. Feigon , and W. L. Hubbell , “Monitoring RNA Base Structure and Dynamics Using Site‐Directed Spin Labeling,” Biochemistry 42 (2003): 6772–6783, 10.1021/bi027222p. 12779332

[27] Y. Toyama , M. Kumihara , K. Takeuchi , and I. Shimada , “NMR Investigation of the Sliding Motion of RNA Helicase DDX3X Along Single‐Stranded RNA,” Biophysical Chemistry 334 (2026): 107615, 10.1016/j.bpc.2026.107615. 41865490

[28] A. Schlundt , J.‐N. Tants , and M. Sattler , “Integrated Structural Biology to Unravel Molecular Mechanisms of Protein‐RNA Recognition,” Methods 118–119 (2017): 119–136, 10.1016/j.ymeth.2017.03.015. 28315749

[29] T. Carlomagno , “Present and Future of NMR for RNA–Protein Complexes: A Perspective of Integrated Structural Biology,” Journal of Magnetic Resonance 241 (2014): 126–136, 10.1016/j.jmr.2013.10.007. 24656085

[30] H. Busch , M. Yasir Ateeque , F. Taube , T. Wiegand , B. Corzilius , and G. Künze , “Probing Biomolecular Interactions With Paramagnetic Nuclear Magnetic Resonance Spectroscopy,” Chembiochem 26 (2025): e202400903. 10.1002/cbic.202400903 PMC11907393 39803829

[31] F. Delaglio , S. Grzesiek , G. W. Vuister , G. Zhu , J. Pfeifer , and A. Bax , “NMRPipe: A Multidimensional Spectral Processing System Based on UNIX Pipes,” Journal of Biomolecular NMR 6 (1995): 277–293, 10.1007/BF00197809. 8520220

[32] M. W. Maciejewski , A. D. Schuyler , M. R. Gryk , et al., “NMRbox: A Resource for Biomolecular NMR Computation,” Biophysical Journal 112 (2017): 1529–1534, 10.1016/j.bpj.2017.03.011. PMC5406371 28445744

