# Supplementary material for: An Electrophilic Uridine Building Block for Post‐Synthetic RNA Modification as Exemplified for Spin Labeling
Source: Chemistry. 2026 Jul 3;32(38):e71319. doi: 10.1002/chem.71319 (PMC13660491; doi:10.1002/chem.71319)
Supplement: Supplementary file 1 [file CHEM-32-e71319-s001.docx]

**SUPPLEMENTARY INFORMATION**

**An Electrophilic Uridine Building Block for Post-Synthetic RNA Modification as Exemplified for Spin Labeling**

Lisa Ruetz^1^, Sonja Leiner^1^, Alessandro Marotto^1^, Raphael Plangger^1^, and Christoph Kreutz^*,1^

^1^ Institute of Organic Chemistry and Center for Molecular Biosciences Innsbruck (CMBI), University of Innsbruck, Innrain 80/82, 6020 Innsbruck, Austria.

* To whom correspondence should be addressed. Email: christoph.kreutz@uibk.ac.at

**Supplementary methods**

**EXPERIMENTAL**

**General.** Chemical reagents and solvents were purchased in the highest available quality from commercial suppliers (Merck/Sigma-Aldrich, ABCR) and used without further purification. Analytical thin-layer chromatography (TLC) was performed on Macherey-Nagel Polygram® SIL G/UV254 plates. 0.2 mm silica gel 60 for column chromatography was purchased from Macherey-Nagel. ^1^H and ^13^C NMR spectra were recorded on a Bruker Ultrashield 400 MHz Avance Neo NMR. Chemical shifts (δ) are reported relative to tetramethylsilane (TMS), referenced to the residual solvent signal (DMSO-d_6_: 2.50 ppm for ^1^H NMR and 39.52 ppm for ^13^C NMR spectra; CDCl_3_: 7.26 ppm for ^1^H NMR and 77.16 ppm for ^13^C NMR spectra). Signal assignments are based on ^1^H-^1^H-COSY, ^1^H-^13^C-HSQC and ^1^H-^13^C-HMBC experiments. High-resolution mass spectra were recorded in positive ion mode unless otherwise noted on a Thermo Scientific Q Exactive Orbitrap.

**Synthesis of 5-ITC-U phosphoramidite 10.**

5-(4,4'-Dimethoxytrityl)oxymethyl-2',3',5'-tribenzoyluridine (**2**)

Compound **1^[^**^1]^ (2.40 g, 4.09 mmol, 1. Eq.) was co-evaporated three times with dry pyridine and then dissolved in 25 ml anhydrous pyridine under argon atmosphere. Then, 4,4'-dimethoxytrityl chloride (DMT-Cl, 1.80 g, 5.32 mmol; 1.3 Eq.) and 4-dimethylaminopyridine (DMAP, 50.0 mg, 409 μmol, 0.1 eq.) were added. The light orange solution was stirred for 2 hours at room temperature. The reaction was quenched with methanol and the solvent was evaporated. The residue was dissolved in ethyl acetate and washed three times with 5% aqueous citric acid solution and once with saturated sodium bicarbonate solution. The acidic and the basic aqueous layers were separately extracted with ethyl acetate. The combined organic layers were dried over sodium sulfate, filtered and evaporated to dryness. The crude product was purified by flash column chromatography (SiO_2_, 7/3 *n-*Hex/EA →1/1 *n*-Hex/EA) to give compound **2**.

Yield: 3.45 g (95%; 3.89 mmol)

TLC: 6/4 *n-*Hex/EA; R*_f_* = 0.3

^1^H-NMR (300 MHz, DMSO-d_6_, 25 °C): δ 11.60 (s, 1H, NH); 8.00-7.41 (m, 15H, benzoyl arom. CH); 7.84 (s, 1H, C(6)H); 7.41-7.16 (m, 9H, DMT arom. CH); 6.87 (m, 4H, 4x DMT arom. CH-C-OCH_3_); 6.25 (m, 1H, C(1’)H); 5.99 (m, 2H, C(2’)H, C(3’)H); 4.79 (m, 1H, C(4’)H); 4.71 (m, 2H, C^5^-CH_2_-O); 3.73 (s, 6H, 2x O-CH_3_); 3.71 (m, 2H, C(5’)H, C(5’’)H) ppm.

ESI-MS (m/z): [M+Na]^+^ calc. 911.28; found 911.28. [M+K]^+^ calc. 927.25; found 927.25.

5-(4,4'-Dimethoxytrityl)oxymethyluridine (**3**)

Compound **2** (9.79, 11.01 mmol, 1.00 Eq.) was dissolved in 70 mL methylamine in EtOH (33%) and stirred at room temperature overnight. The solution was evaporated and crude compound **3** purified using column chromatography (SiO_2_, 96/4 – 92/8 DCM/MeOH).

Yield: 6.05 g (10.49 mmol, 95%)

TLC: (9/1: DCM/MeOH); R_f_ = 0.5

^1^H-NMR (400 MHz, DMSO-d_6_, 25 °C): δ 11.41 (s, 1H, N*H*); 7.85 (s, 1H, C(6)*H*); 7.41-6.89 (m, 13H, arom. C*H*); 5.87 (d, 1H, C(1’)*H*, ^3^J_HH_ = 6,18 Hz); 5.39 (d, 1H, C(2’)O*H*, ^3^J_HH_ = 6.13 Hz); 5.14 (d, 1H, C(3’)O*H*, ^3^J_HH_ = 5.27 Hz); 5.02 (t, 1H, C(5’)O*H*, ^3^J_HH_ = 5.21 Hz); 4.10 (q, 1H, C(2’)*H*, ^3^J_HH_ = 5.62 Hz); 4.00 (m, 1H, C(3’)*H*); 3.89 (m, 1H, C(4’)*H*); 3.75 (s, 6H, 2x OC*H*_3_); 3.73 (s, 2H, C^5^-C*H*_2_-O); 3.62 (m, C(5’)*H*, C(5’’)*H*).

2'-*O*-(*tert*-butyldimethylsilyl)-3',5'-*O*-(di-*tert*-butyl)silyl-5-(4,4'-dimethoxytrityl)oxymethyluridine (**4**)

Compound **3** (6.05 g, 10.49 mmol, 1.00 Eq.) was dissolved in 25 mL anhydrous DMF under argon atmosphere. Pyridine (8.47 mL, 8.30 g, 104.93 mmol, 10.00 Eq.) was added and the mixture cooled to 0 °C using an ice bath. Di-*tert*-butylsilylbis(trifluoromethanesulfonate) (3.73 mL, 5.08 g, 11.54 mmol, 2.50 Eq.) was added dropwise. Imidazole (1.79 g, 26.23 mmol, 2.50 Eq.) and *tert*-butyldimethylsilyl chloride (1.90 g; 12.59 mmol. 1.20 Eq.) were added and the mixture heated to 60 °C and stirred for 3h. The reaction was quenched by the addition of MeOH. The solvent was evaporated, the residue dissolved in ethyl acetate, washed with 5%-citric acid, saturated sodium bicarbonate solution and saturated sodium chloride solution. The organic layer was dried over sodium sulfate and the filtrate evaporated yielding crude compound **4**.

Yield: 8.69 g (10.49 mmol, 95%)

TLC: (97/3: DCM/MeOH); R_f_ = 0.8

^1^H-NMR (400 MHz, DMSO-d_6_, 25 °C): δ = 11.47 (s, 1H, N*H*); 7.50 (s, 1H, C(6)*H*); 7.39-6.81 (m, 13H, arom. C*H*); 5.69 (s, 1H, C(1’)*H*); 4.57 (m, 1H, C(5’)*H*); 4.45 (d, 1H, C(2’)*H*, ^3^J_HH_ = 3.62 Hz); 4.05-3.93 (m, 3H, C(3’)*H*,C(4’)*H*, C(5’’)*H*); 3.74 (s, 2H, C^5^-C*H*_2_-O); 3.72 (s, 6H, 2x OC*H*_3_); 1.01-0.89 (3s, 27H, 3x Si-C-(C*H*_3_)_3_); 0.17-0.07 (2s, 6H, 2’-Si-(C*H*_3_)_2_).

2'-*O*-(*tert*-butyldimethylsilyl)-3',5'-*O*-(di-*tert*-butyl)-silyl-5-hydroxymethyluridine (**5**)

Compound **4** (8.69 g, 10.49 mmol) was dissolved in 200 mL anhydrous dichloromethane. Dichloroacetic acid (8.64 mL, 13.48 g, 104.55 mmol, 10.00 Eq.) and pyridine (3.62 mL, 3.51 g, 52.28 mmol, 5.00 Eq.) were added and the solution stirred at room temperature for 30 minutes under argon atmosphere. The mixture was diluted with dichloromethane and washed with half-saturated sodium bicarbonate solution. The organic layer was dried over sodium sulfate and the filtrate evaporated. Crude compound **5** was purified by column chromatography (SiO_2_, 3/7 -1/1 EA/*n*-Hex).

Yield: 3.09 g (5.84 mmol, 56%)

TLC: (4/6: EA/*n*-Hex); R_f_ = 0.25

^1^H-NMR (400 MHz, DMSO-d_6_, 25 °C): δ = 11.44 (s, 1H, N*H*); 7.39 (s, 1H, C(6)*H*); 5,70 (s, 1H, C(1’)*H*); 4.99 (s, 1H, O*H*); 4.42 (m, 2H, C(2’)*H*, C(4’)*H*); 4.15 (d, 2H, C^5^-C*H*_2_-O, ^3^J_HH_ = 5.43); 4.04 (m, 1H, C(5’)*H*); 3.94 (m, 2H, C(5’’)*H*, C(3’)*H*); 1.05-0.88 (3s, 27H 3x Si-C(C*H*_3_)_3_); 0.11 (2s, 6H, 2x Si-C*H*_3_).

2'-*O*-(*tert*-butyldimethylsilyl)-3',5'-*O*-(di-*tert*-butyl)silyl-5-(pivaloyloxy)methyluridine (**6**)

Compound **5** (3.09 g, 5.84 mmol, 1.00 Eq.) was dissolved in 40 mL anhydrous pyridine under argon atmosphere. Pivaloyl chloride (0.94 mL, 916 mg, 7.60 mmol, 1.30 Eq.) was added and the reaction mixture stirred at RT for two and a half hours. The solvent was evaporated, the residue dissolved in ethyl acetate and washed with 5% aqueous citric acid and saturated sodium bicarbonate solution. The organic layer was dried over sodium sulfate and the filtrate evaporated. Crude compound **6** was purified by column chromatography (SiO_2_, 1/9 – 3/7 EA/*n*-Hex).

Yield: 3.35 g (5.47 mmol, 94%)

TLC: (4/6: EA/*n*-Hex); R_f_ = 0.85

^1^H-NMR (400 MHz, DMSO-d_6_, 25 °C): δ = 11.59 (s, 1H, N*H*); 7.65 (s, 1H, C(6)*H*); 5.65 (s, 1H, C(1’)*H*); 4.73 (m, 2H, C(2’)*H*, C(4’)*H*); 4.42 (d, 2H, C^5^-C*H*_2_-O, ^3^J_HH_ = 4.67); 4.06-3.93 (m, 3H, C(5’)*H*, C(5’’)*H*, C(3’)*H*); 3.31 (s, 9H, piv. C(C*H*_3_)_3_); 1.12-0.98 (3s, 27H, 3x Si-C(C*H*_3_)_3_,); 0.14-0.09 (2s, 6H, 2x Si-C*H*_3_).

2'-*O*-(*tert*-butylmethylsilyl)-3',5'-*O*-(di-*tert*-butyl)-silyl-5-(amino)methyluridine (**7**)

Compound **6** (3.35, 5.47 mmol, 1.00 Eq.) was dissolved in 70 mL ammonia in MeOH (7 mol/l) in a pressure tube. The reaction mixture was stirred at 70 °C overnight. The solvent was evaporated, the residue dissolved in ethyl acetate and washed with saturate sodium bicarbonate solution. The organic layer was dried over sodium sulfate and the filtrate evaporated. Crude compound **7** was purified by column chromatography (SiO_2_, 10/0 – 9/1 DCM/MeOH).

Yield: 1.47 g (2.79 mmol, 51%)

TLC: (9/1: DCM/MeOH); R_f_ = 0.30

^1^H-NMR (400 MHz, DMSO-d_6_, 25 °C): δ = 7.39 (s, 1H, C(6)*H*); 5.72 (s, 1H, C(1’)*H*); 4.39 (m, 2H, C(2’)*H*, C(5’)*H*); 4.08 (d x d, 1H, C(3’)*H*, ^3^J_HH_ = 5.00), 4.00 (t, 1H, C(5’’)*H*), 3.86 (m, 1H, C(4’)*H*); 3.34 (s, 2H, C*H*_2_) 1.05-0.86 (3s, 27H, 3x Si-C(C*H*_3_)_3_,); 0.13-0.08 (2s, 6H, 2x Si-C*H*_3_).

2'-*O*-(*tert*-butyldimethylsilyl)-3',5'-*O*-(di-*tert*-butyl)silyl-5-(isothiocyanato)methyluridine (**8**)

Compound **7** (400 mg, 785 mmol, 1.00 Eq.) was dissolved in 15 mL anhydrous chloroform under argon atm. Thiophosgene (0.377 μL, 122 mg, 1.06 μmol, 1.40 Eq.) was diluted with 4 mL anhydrous chloroform and the solution added dropwise to the mixture and the reaction mixture stirred for 3 hours at room temperature. Afterwards the mixture was diluted with dichloromethane and washed with saturated sodium bicarbonate solution. The organic layer was dried over sodium sulfate and the filtrate evaporated. Crude compound **8** was purified by column chromatography (SiO_2_, 1/9 – 1/1 EA/*n*-Hex).

Yield: 370 mg (649 μmol, 86%)

TLC: (9/1: DCM/MeOH); R_f_ = 0.30

^1^H-NMR (400 MHz, CDCl_3_, 25 °C): δ = 8.89 (s, 1 H, N*H*); 7.40 (s, 1H, C(6)*H*); 5.74 (s, 1H, C(1’)*H*); 4.39 (m, 2H, C(2’)*H*, C(5’)*H*); 4.09-3.86 (m, 3H, C(3’)*H*, C(4’)*H*, C(5’’)*H*); 3.34 (s, 2H, C*H*_2_); 1.09-0.92 (3s, 27H, 3x Si-C(C*H*_3_)_3_); 0.20-0.14 (2s, 6H, 2x Si-C*H*_3_).

2'-*O*-(*tert*-butyldimethylsilyl)-5'-*O*-(4,4'-dimethoxytrityl)-5-(thiocyanate)methyluridine (**9**)

Compound 8 (920 mg, 1.61 mmol, 1.00 Eq.) was dissolved in 8.6 mL anhydrous dichloromethane under argon atmosphere. Hydrogen fluoride (112.73 μL, 124 mg, 6.22 mmol, 3.85 Eq.) was diluted with pyridine (1.04 mL, 1.02 g, 12.9 mmol, 8.00 Eq.) and added to the reaction mixture. The mixture was stirred for 2 hours and 30 minutes. The solvent was evaporated to give the crude compound.

Yield: quantitative (694 mg, 1.61 mmol)

TLC: (95/5: DCM/MeOH); R_f_ = 0.30

The crude compound (694 mg, 1.61 mmol, 1.00 Eq.) from the previous step was co-evaporated with pyridine and then dissolved in 20 mL anhydrous pyridine under argon atmosphere. Then, 4,4'-dimethoxytrityl chloride (DMT-Cl, 821 mg, 2.42 mmol, 1.50 Eq.) was added in three portions over the course of an hour. The mixture was stirred at room temperature overnight. The solvent was evaporated, the residue dissolved in ethyl acetate and washed with 5% aqueous citric acid and saturated sodium bicarbonate solution. The organic layer was dried over sodium sulfate and the filtrate evaporated. Crude compound **9** was purified by column chromatography (SiO_2_, 1/9 – 4/6 EA/*n*-Hex).

Yield: 1.14 g (1.56 mmol, 96%)

TLC: (95/5: DCM/MeOH); R_f_ = 0.8

^1^H-NMR (400 MHz, DMSO-d_6_, 25 °C): δ = 8.01 (s, 1H, N*H*); 6.03 (d, 1H,C(6)*H*); 4.47 (m, 1H, C(1’)*H*); 4.18-4.10 (m, 2H, C(2’)*H*, C(5’)*H*); 3.54 (m, 2H, C*H*_2_); 3.47-3.19 (m, 2H, C(3’)*H*, C(4’)*H*); 2.68 (m, 1H, C(5’’)*H*); 1.56-1.26 (m, 19H, DMT-O-); 0.94 (s, 9H, Si-C(CH_3_)_3_); 0.16 (d, 6H, Si- CH_3_).

2'-O-tert-butyldimethylsilyl-3'-O-(2-cyanoethyl-N,N-diisopropyl-phosphoramidite)-5'-O-(4,4'-dimethoxytrityl)-5-thiocyanatemethyluridine (**10**)

Compound **9** (750 mg, 1.02 mmol, 1.00 Eq.) was co-evaporated with tetrahydrofuran and then dissolved in 10 mL anhydrous tetrahydrofuran under argon atmosphere. Diisopropylethylamine (DIPEA, 727 mg, 3.07 mmol, 3.00 Eq.) and 2-cyanoethyl *N*,*N*-diisopropylchloro phosphoramidite (CEP-Cl, 397 mg, 10.25 mmol, 10.00 Eq.) were added and the solution stirred at room temperature for one hour. The reaction mixture was diluted with dichloromethane and washed with half saturated sodium bicarbonate solution. The organic layer was dried over sodium sulfate and the filtrate evaporated. Crude compound **10** was purified by column chromatography (SiO_2_, 2/8 – 4/6 EA/*n*-Hex + 1% triethylamine).

Yield: 490 mg (526 μmol, 51%)

TLC: (7/3: *n*-Hex/EA); R_f_ = 0.4

^1^H-NMR (400 MHz, DMSO-d_6_, 25 °C): δ = 8.04 (m, 1H, C(6)*H*); 7.40-7.23 (m, 9H; arom. *H*); 6.90-6.83 (s, 4H, arom. *H*); 6.01 (m, 1H, C(1’)*H*); 4.51- 3.40 (m, 5H, C(2’)*H*, C(3’)*H*, C(4’)*H*, C(5’)*H*, C(5’’)*H*); 3.36 (m, 2H, C*H*_2_); 1.32-1.12 (m, 12H, N-C-C*H*_3_); 1.07 (m, 4H, C*H*_2_), 0.90 (m, 9H, Si-C(C*H*_3_)_3_); 0.14 (m, 6H, Si-C*H*_3_).

^31^P-NMR (162 MHz, DMSO-d_6_, 25°C): δ = 150.17 (s); 149.84 (s).

ESI-MS (m/z): calc.: 931.38, found 932.38 [M+H]^+^, 954.36 [M+Na]^+^, 970,33 [M+K]^+^.

**Supporting Figure 1.** ^1^H-NMR of 5-isothiocyanatomethyluridine phosphoramidite **10** (400 MHz, DMSO-d_6_, 25 °C).

**Supporting Figure 2. ^3^**^1^P-NMR of 5-isothiocyanatemethyluridine phosphoramidite **10** (400 MHz, DMSO-d_6_, 25 °C). **A)** Full spectral width and **B)** Zoom on the ^31^P resonances of **10**.

**Chemical synthesis of spin and stable isotope labelled RNAs**

Standard 2’-*O*-TBDMS RNA phosphoramidites (rA^Ac^, rC^Ac^, rG^Ac^ and rU, *Chemgenes*, USA) were used in combination with in-house synthesized [5-D-6-^13^C]-uridine, [5-D-6-^13^C]-cytidine, [1,3-^15^N_2_]-uridine, [1-^15^N]-adenosine and the respectively 5-isothiocyanatomethyluridine modified RNA phosphoramidite. Controlled pore glass (CPG) RNA solid support (1000 Å pore size, *ChemGenes*, USA) with an average loading of 40 µmol g^-1^ was used to synthesize the RNAs on an ABI 391 PCR Mate using a self-written RNA synthesis cycle. Amidite (0.1 M) and activator (5-benzylthio-*1H*-tetrazole, 0.25 M) solutions were dried over freshly activated molecular sieves (3 Å) for at least 48 hours. The following reagent mixtures were used: *Cap A*: acetic anhydride/lutidine/tetrahydrofuran 1/1/8, v/v/v. *Cap B*: tetrahydrofuran/N-methylimidazole 86/16 v/v. *Oxidation solution*: 500 mg iodine dissolved in a mixture of 70 mL THF, 20 mL pyridine and 10 mL water. *Detritylation solution*: 4% dichloroacetic acid in anhydrous toluene.

After the complete RNA synthesis, the solid support was dried for 1h in high vacuum. The dried solid support was treated with 1.4 mL of 50 mM 4-Amino-TEMPO in DMSO for 24h at room temperature.

Standard a*lkaline deprotection*: 1 mL methylamine (40%) in water and 1 mL ammonia (28%) in water were added to the solid support. The reaction tube was shaken vigorously and incubated at 37°C for 5 hours. The solid support was pelleted via centrifugation, and the supernatant was transferred to a 10 mL round bottom flask. The remaining solid support was washed three times with a mixture of THF/water (1/1), the liquid phases were combined with the first filtrate and evaporated to dryness. The residual white precipitate was dried in high vacuum for at least 1h. *2’-O-TBDMS deprotection:* The residue from the previous step was dissolved in 300 µL anhydrous dimethyl sulfoxide, and triethylamine trihydrofluoride was added (50 eq. per TBDMS group) and the deprotection mixture incubated at 37°C for at least 16h. Then, the deprotection mixture was quenched with 2 mL quenching buffer (*GlenResearch*, USA) and directly applied to a HiPrep 26/10 desalting column (*GE Healthcare*, Austria) using a ÄKTA start system (*GE Healthcare*, Austria). The crude RNA was eluted using HPLC grade water and the RNA containing fractions (UV detection at 254 nm) were collected in a 50 mL round bottom flask. After evaporation, the crude RNA was dissolved in 1 mL HPLC grade water and transferred to a 1.5 mL reaction tube. The crude RNA was stored at -20°C. The quality of the crude RNAs was checked via anion exchange chromatography on an analytical Dionex DNAPac PA-200 column (4x250 mm; *Eluent A*: 25 mM Tris.HCl, 10 mM sodium perchlorate, 20 % acetonitrile, pH 8.0; *Eluent B*: 25 mM Tris.HCl, 600 mM sodium perchlorate, 20 % acetonitrile, pH 8.0) and at elevated temperature (80 °C). Purification of the RNA sequences was achieved in a single run by applying the crude RNA to a preparative Dionex DNAPac PA-200 column (22x250 mm, eluents as before). The fractions containing the desired RNA were pooled and loaded on a C18 SepPak cartridge (*Waters*, Austria) to remove HPLC buffer salts. The RNA sodium salt form was then eluted from the C18 column with water/acetonitrile (1/1, v/v), concentrated and transferred to a 1.5 ml reaction tube for concentration determination and mass spectrometric analysis. Sample concentrations were determined by measuring UV absorption at 260 nm on a NanoPhotometer (*Implen*).

**LC-ESI mass spectrometry.** All RNAs were analyzed on Finnigan LCQ Advantage MAX ion trap instrumentation connected to a Thermo Scientific UHPLC (components: Ultimate 3000 RS Pump, Ultimate 3000 RS Autosampler, Ultimate 3000 RS Column Compartment, Ultimate 3000 Diode Array Detector). RNA mass spectra were acquired in the negative-ion mode with a potential of -4 kV applied to the spray needle (capillary temperature: 270°C, capillary voltage: -23V). LC: 250 pmol RNA dissolved in 30 μl of 20 mM ethylenediaminetetraacetic acid (EDTA) solution; average injection volume: 30 μl; column: Waters xBridge C18 2.5µm column (1.0 × 50 mm) at 30°C; flow rate: 100 μL/min; Eluent A: 8.6 mM triethylamine (TEA), 100 mM 1,1,1,3,3,3-hexafluoroisopropanol in H_2_O (pH 8.0); Eluent B: methanol; gradient: 0–100% B in A within 30 min; UV detection at 260/280 nm. The correct assembly of all RNAs used in this study was confirmed by the mass data.

**NMR spectroscopy.** RNA samples were lyophilized as sodium salts and dissolved in 450 µL NMR buffer (15 mM sodium phosphate, 25 mM NaCl, 0.1% NaN_3_, 0.1 mM Na EDTA, pH 6.5, 10 % D_2_O, 298 K) and transferred into standard 5 mm NMR tubes giving ca. 350 µM sample concentrations. All NMR experiments were conducted on a Bruker 600 MHz Avance Neo or a Bruker 700 MHz Avance Neo NMR both equipped with a Prodigy TCI probe. Imino proton spectra were acquired with a selective excitation scheme.

For the quantitative determination of the PRE effect in RNA **12** the Bruker pulse program ‘hsqcetfpgpsi2’ was adapted as proposed by Clore and co-workers.^[2]^ In brief, a variable delay in the first INEPT was introduced and the transverse relaxation rates of the ^13^C-bound H6 resonances in the para- and diamagnetic state were determined in a six point measurement with two repeats with the following relaxation delays: 4, 6 (2x), 8, 10 (2x), 12 and 15 milliseconds. The pseudo 3D spectra were processed and peak fitting was achieved by using the nmrpipe software package^[3]^ provided via the NMRbox server.^[4]^ The ^1^H transverse relaxation rates were determined by fitting the intensities to a mono-exponential decay:

I(t) = I_0_ x exp(-R_2_/t)

Parameter standard errors were taken as the square roots of the diagonal elements of the covariance matrix. Fit quality was monitored via residuals, reduced chi-square, and Akaike Information Criterion (AIC); obvious outliers (e.g., due to overlap) were excluded. Paramagnetic and diamagnetic transverse relaxation rates were defined as R_2,para_ and R_2,dia_, respectively, determined as above from matched residues. The PRE rate was calculated as:

^1^H-Γ_2_ = R_2,para_ – R_2,dia_

The uncertainty in ΔR_2_ assumed independent errors and was propagated by quadrature:

d(ΔR_2_) = [(dR_2,para_)^2^+(dR_2,dia_)^2^]^1/2^

Reported values are ΔR_2_ ± d(ΔR_2_) in s^-1^. Where multiple replicate datasets were available, per-condition R_2_ values were first averaged, and between-replicate standard errors were combined with fit-based errors in quadrature. Python scripts using the packages NumPy, SciPy and Matplotlib were used for fitting and plotting. The relaxation data and the python scripts are available for download ((UIBK data repository, DOI: https://doi.org/10.48323/fmkhq-xf509).

**Supplementary Figures**

**Supporting Figure 3.** Mass spectrometric data of 9 nt RNA sequence after work-up procedure A (**A**) and B (**B**).

**Supporting Figure 4.** Qualitative determination of PRE effect in SLC^A^. **A)** Intensity ratios of long range ^1^H2-^15^N1-correlations of adenosine in RNA **11** from para- and diamagnetic long range SOFAST experiments. The PRE effects are mapped on the SLC^A^ secondary structure. **B)** Intensity ratios of H5-H6 pyrimidine nucleobase correlations in RNA **11** from para- and diamagnetic TOCSY experiments. The PRE effects are mapped on the SLC^A^ secondary structure

**Supporting Figure 5.** Structure model of TEMPO tagged SLC^A^ at U31. Distances to U4 (14.6 Å), U8 (19.6 Å) and C35 (16.9 Å) are shown.

**Supporting Figure 6.** Analytical and spectroscopic data of double TEMPO tagged RNA. **A)** Stable isotope and 2xTEMPO labeling pattern of the SLC^A^ construct. Note: Only 25% ^13^C labelling was used. **B)** ESI-MS data and calculated mass. **C)** ^1^H NMR spectra with ^15^N decoupling and focus on the imino proton region before (red trace) and after (green trace) radical quench with sodium ascorbate. Significant PRE effects were observed for all observable imino proton resonances. **D)** ^1^H-^13^C HSQC spectrum of 2xTEMPO tagged SLC^A^ before (red) and after (green) radical quench with sodium ascorbate**.** Note: The spectra are shifted the in the indirect dimension to easily observe the PRE effect. **E)** EPR spectrum of 2xTEMPO tagged SLC^A^ at 30°C.

**REFERENCES**

[1]	X. Yan, H. Sun, C. Mo, L. Ma, C. Hu, Z. Zhong, Y. Su, L. Yin, J. Zhang, J. Chen, Y. Liu, X. Han, Y. Liu, J. Xu, S. Cen, Y. Chen, X. He, *Bioorganic Chemistry* **2026**, *170*, 109472.

[2]	J. Iwahara, C. D. Schwieters, G. M. Clore, *Journal of the American Chemical Society* **2004**, *126*, 5879-5896.

[3]	F. Delaglio, S. Grzesiek, G. W. Vuister, G. Zhu, J. Pfeifer, A. Bax, *Journal of Biomolecular NMR* **1995**, *6*, 277-293.

[4]	M. W. Maciejewski, A. D. Schuyler, M. R. Gryk, I. I. Moraru, P. R. Romero, E. L. Ulrich, H. R. Eghbalnia, M. Livny, F. Delaglio, J. C. Hoch, *Biophysical Journal* **2017**, *112*, 1529-1534.
